# Social Cognition in Individuals at Ultra-High Risk for Psychosis: A Meta-Analysis

**DOI:** 10.1371/journal.pone.0141075

**Published:** 2015-10-28

**Authors:** R. J. M. van Donkersgoed, L. Wunderink, R. Nieboer, A. Aleman, G. H. M. Pijnenborg

**Affiliations:** 1 Department of Clinical Psychology and Experimental Psychopathology, Faculty of Behavioral and Social Sciences, University of Groningen, Groningen, The Netherlands; 2 Department of Education and Research, Friesland Mental Health Care Services, Leeuwarden, The Netherlands; 3 Department of Neuroscience, BCN Neuroimaging Center, University Medical Center Groningen, Groningen, The Netherlands; 4 Department of Psychotic Disorders GGZ-Drenthe, Assen, The Netherlands; Maastricht University, NETHERLANDS

## Abstract

**Objective:**

Treatment in the ultra-high risk stage for a psychotic episode is critical to the course of symptoms. Markers for the development of psychosis have been studied, to optimize the detection of people at risk of psychosis. One possible marker for the transition to psychosis is social cognition. To estimate effect sizes for social cognition based on a quantitative integration of the published evidence, we conducted a meta-analysis of social cognitive performance in people at ultra high risk (UHR).

**Methods:**

A literature search (1970-July 2015) was performed in PubMed, PsychINFO, Medline, Embase, and ISI Web of Science, using the search terms ‘social cognition’, ‘theory of mind’, ‘emotion recognition’, ‘attributional style’, ‘social knowledge’, ‘social perception’, ‘empathy’, ‘at risk mental state’, ‘clinical high risk’, ‘psychosis prodrome’, and ‘ultra high risk’. The pooled effect size (Cohen’s D) and the effect sizes for each domain of social cognition were calculated. A random effects model with 95% confidence intervals was used.

**Results:**

Seventeen studies were included in the analysis. The overall significant effect was of medium magnitude (d = 0.52, 95% Cl = 0.38–0.65). No moderator effects were found for age, gender and sample size. Sub-analyses demonstrated that individuals in the UHR phase show significant moderate deficits in affect recognition and affect discrimination in faces as well as in voices and in verbal Theory of Mind (TOM). Due to an insufficient amount of studies, we did not calculate an effect size for attributional bias and social perception/ knowledge. A majority of studies did not find a correlation between social cognition deficits and transition to psychosis, which may suggest that social cognition in general is not a useful marker for the development of psychosis. However some studies suggest the possible predictive value of verbal TOM and the recognition of specific emotions in faces for the transition into psychosis. More research is needed on these subjects.

**Conclusion:**

The published literature indicates consistent general impairments in social cognition in people in the UHR phase, but only very specific impairments seem to predict transition to psychosis.

## Introduction

Early signs of psychosis can be present several years before the actual onset of illness. Many individuals show a decline in social functioning before onset [1]. Other signs that may occur prior to onset are mild positive symptoms, negative symptoms such as social withdrawal, depressive symptoms and basic symptoms such as subclinical self-experienced disturbances in thought, speech and perception processes [2].

People at heightened risk for psychosis, usually referred to as Clinical high risk (CHR), Ultra high risk (UHR), psychosis prodrome or At Risk Mental State (ARMS), meet the following criteria: presence of attenuated psychotic symptoms (APS) and/or a family history of schizophrenia combined with problems in functioning [3, 4] and/or presence of one or more brief limited intermittent psychotic symptoms (BLIPS) such as delusions or hallucinations [5]. A recent meta-analysis shows that the average 1-year transition rate to psychosis in this UHR group was 22%, increasing to 36% after three years follow-up [6].

Basic symptoms (BS) where suggested as an alternative set of criteria to detect at risk patients [7]. Basic symptoms consist of subclinical subtle disturbances in stress tolerance, affect, thinking, speech, drive, perception and motor action [8, 9] appearing before the appearance of APS or BLIPS, thus allowing for detection of at risk patients at an earlier stage. There is accumulating evidence that the onset of psychosis can be prevented by intervening in this risk phase [10–12] and treatment in the early stages of schizophrenia is critical to the progression of the disease [13–15].

Given the high number of false positives, it is difficult to predict which individuals in the UHR group will develop psychosis on the basis of their clinical features [16]. Therefore there is a clear need for better prediction models that can be used to help clinicians identify a subgroup of subjects that will benefit most from preventive interventions [17]. In the past decade markers for the development of psychosis have been studied, to optimize the detection of people in the prodromal phase. Several clinical models have been proposed to further increase the validity of prediction of transition to psychosis in the UHR group [18, 19].

One possible marker of a liability for psychosis is social cognition [20], which is defined as the perception, interpretation and processing of social information [21]. Impaired social cognition is considered to result in poor social functioning, a well-established risk factor for transition to psychosis [22]. Expert surveys identified four core domains of social cognition in schizophrenia research [23]: emotional perception and processing, in particular the recognition of facial and vocal affect [24, 25], social perception and knowledge, such as decoding of non-verbal communication and social cue recognition [26], theory of mind (TOM), the mental capacity to infer ones own and other mental states, with subdomains verbal and visual TOM [27, 28] and attributional style, the tendency to attribute the cause of events to the self, others or the environment [29, 30].

Impairments in all of these domains have been consistently found in the acute phase of psychosis as well as in the remission phase [31] and in patients with chronic schizophrenia as well as patients with first onset psychosis [32]. Furthermore, a recent meta-analysis found that adult first degree relatives of people with schizophrenia show moderate difficulties in social cognition [33] and a recent meta-analysis on theory of mind in participants in the UHR phase showed significant, moderate effect sizes [34].

In the current meta-analysis we aimed to investigate whether performance on measures of social cognition is already impaired in the at risk phase. We limited our analyses to studies that used the UHR criteria for inclusion, as most studies on social cognition in the at risk phase use these criteria and inclusion of both UHR and BS may yield an overly heterogeneous sample and hence complicate interpretation.

Two previous reviews on social cognition and at risk participants were found. Thompson et al. [20] used a qualitative approach and reviewed seven studies (published before 2011) on social cognition in people at clinical ultra high risk of psychosis, concluding that deficits in social cognitive abilities do appear to be present in the UHR population. Lee et al. [35] performed a meta-analysis on social cognition in UHR patients and BS patients taken together as one group. They investigated the four general domains of social cognition as stated by Pinkham et al. [23], and found that deficits in social cognitive abilities are present in the at risk population.

Our meta-analysis adds to the previous one by selecting a more homogeneous sample and by conducting additional, more fine-grained analyses to the four domains of social cognition by examining whether specific impairments occur for prosodic or facial affect recognition and verbal or visual TOM. We also include three additional studies that appeared after the meta-analysis by Lee et al. [36–38] and we conduct a comprehensive qualitative evaluation of social cognition and transition to psychosis. Finally, we investigate whether potential moderators (age, sex, IQ, sample size) influence general social cognition in UHR participants.

## Methods

### Selection of studies

A literature search was performed by three independent researchers (RD, RN, MP) in PubMed, PsychINFO, Medline, Embase, Picarta and ISI Web of Science published between January 1970 and July 2015, using the following search terms in all possible combinations: ‘social cognition’, ‘theory of mind’, ‘emotion recognition’, ‘attributional style’, ‘social knowledge’, ‘social perception’, ‘empathy’, ‘at risk mental state’, ‘clinical high risk’, ‘psychosis prodrome’, and ‘ultra high risk’. A first selection was made based on the title of the studies followed by a second selection based on the abstract of the remaining articles. Lastly the full articles were examined to determine the final selection to be included into the meta-analysis. A cross-reference search of eligible articles was conducted to identify additional studies not found in the electronic search.

### Inclusion criteria

Inclusion criteria were: a) UHR was defined in line with the criteria of Yung and McGorry [3], b) individuals in the UHR phase were help-seeking, c) a healthy control group was included, d) at least one social cognitive test was administered. For the research question regarding transition into psychosis, we used the same criteria, but we did not exclude studies without a healthy control group.

### Statistical analysis

An effect size was computed for each social cognitive test result, using software developed by Wilson [39]. For each study, one effect size was calculated for each cognitive domain: when multiple tests for the same social cognitive domain were used, the results were combined into one mean effect to ensure all studies contributed equally to the total effect size of the social cognitive domain. The analyses where performed with the program ‘Review Manager 5.0’, developed by the Cochrane Collaboration. Effect sizes where weighted by their standard error to ensure that larger studies had more weight in the mean effect size across studies. Because of the variance in measurements and because the underlying concepts are not unequivocal, a random effects model was used. Overall effect size was calculated and represented in a forest plot, as well as the effect size of each study. Mean effect size, standard error, 95% confidence interval, corresponding z-value and significance level are reported. Variability in outcome of studies caused by clinical and methodological diversity of the studies was tested with Chi-square tests. We performed an Egger’s test [40] to investigate whether publication bias was present, using a regression analysis with the standard normal deviate (Cohen’s D/SE) as a dependent variable and the precision (1/SE) as an independent variable.

We planned to evaluate the potential moderating influence on effect size of age and gender using categorical models (Chi square). These factors were chosen given that social cognition was associated with age, gender an IQ in previous studies [41–43]. Unfortunately, the moderating effect of IQ could not be calculated as not enough data were provided in the articles. We investigated sample size as an additional moderator as smaller samples provide a greater risk of finding a false positive result. We separated the included studies in two groups based on each factor, calculated the mean effect size of each group and tested the significance of the difference between the mean effect sizes using ANOVA-analysis.

## Results

### Literature search

**Fig 1:**
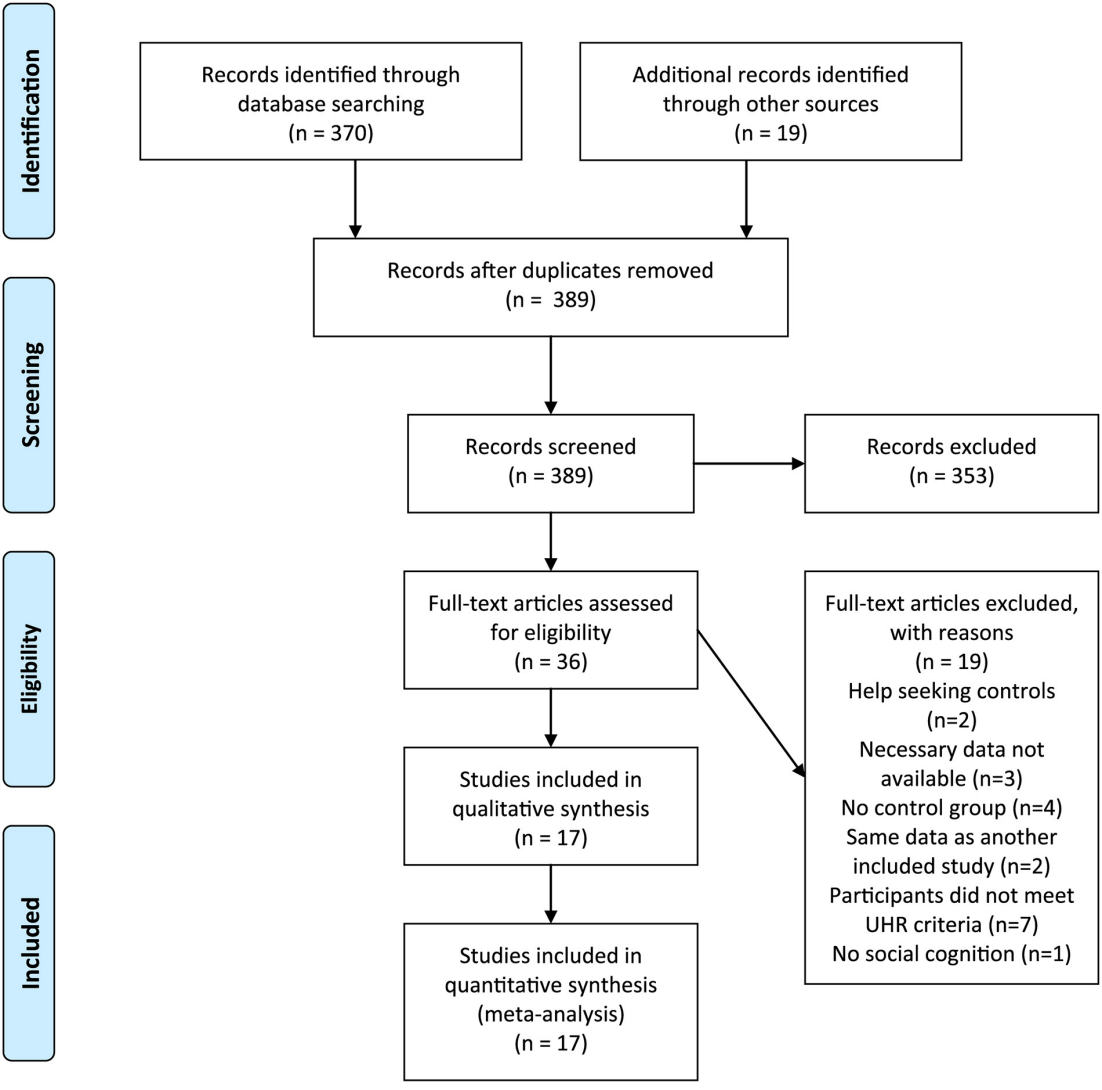
Flow diagram of the literature search. PRISMA flow diagram for meta-analysis of social cognition in individuals at ultra-high risk of psychosis. *From*: Moher D, Liberati A, Tetzlaff J, Altman DG, The PRISMA Group (2009). Preferred Reporting Items for Systematic Reviews and Meta-Analyses: The PRISMA Statement. PLoS Med 6(6): e1000097. doi:10.1371/journal.pmed1000097. For more information, visit www.prisma-statement.org.

The literature search based on title resulted in 389 abstracts. See Fig 1 for a flow diagram of the search. After examination, most of the selected studies where excluded because they did not report on social cognition in individuals in the UHR phase. Thirty-six studies met the inclusion criteria based on the abstract. With further examination of the full text, two more studies were excluded because the control group consisted of help seeking controls instead of healthy controls [44, 45]. Three studies were omitted because necessary data for meta-analysis (mean and standard deviation of the separate groups or change scores) were not provided in the paper, and could not be obtained upon request [46–48]. Four studies were excluded for the first research questions due to a research design without a control group [49–52]. One of these studies [49] reported on conversion to psychosis and was therefore included in the analysis of the possible prediction of psychosis with social cognition. Two studies [53, 54] were excluded because they reported on the same data as another included study [55, 56], we included the most recent studies, since they provided the necessary data and included more participants. Seven more studies were omitted because the included participants did not meet the UHR criteria for this analysis: two studies selected the UHR participants based on cognitive disturbances [57, 58]; another study included participants with familial high risk without functional decline [59]; one study included all help-seeking individuals without selection based on symptoms [60]; one study included schizophrenia patients and no at risk group [61]; one study included participants with a history of psychosis in the at risk group [62] and one study included non-help seeking persons in the UHR group [63]. Finally, one more article was excluded because it examined the processing of facial features (the ability of participants to discriminate between different faces) instead of the recognition of affect in faces [64]. Eventually, seventeen studies were selected for this analysis. All studies were published between 2006 and 2015 and a total of 793 individuals in the UHR phase and 630 in the control condition were included. For an overview of the included studies, see Table 1.

**Table 1:** Overview of included studies.

Reference	n(UHR)	Criteria	Other groups	Assessments	Subgroup
Addington et al., 2008	86	SIPS	Chron, FEP	FEIT, FEDT	AR
Amminger et al., 2012	79	CAARMS	FEP	Facial, Prosodic	AR
An et al., 2010	24	SIPS	FEP	Hostility, Blame	AS
Comparelli et al., 2013	43	SIPS	Chron, FEP	Identification, Recognition	AR
Corcoran et al., 2015	49	SIPS	None	EMODIFF, Audio recordings	AR
Corigliano et al., 2014	36	SIPS	Chron, FEP	Facial affect recognition task	AR
Couture et al., 2008	88	SIPS	Early sz < 5jr	Eyes, ATT	TOM, SP
DeVylder et al., 2013	33	SIPS	None	IPSAQ	AS
Gill et al., 2014	60	SIPS	None	Video Social Inference task	TOM
Green et al., 2011	50	SIPS	Chron, FEP	MSCEIT. TASIT, RAD	AR, TOM, SP
Hur et al., 2013	55	CAARMS	None	False Belief, Strange Story, Cartoon	TOM
Lee et al., 2015	40	SIPS	FEP	Facial emotion recognition task	AR
Pinkham et al., 2007	19	SIPS	Chron, FEP	FEIT, FEDT	AR
Seifert et al., 2008	12	SIPS	None	Discrimination task	AR
Stanford et al., 2011	63	SIPS	Chron	Strange Story, Eyes	TOM
Szily et al., 2009	26	CAARMS	Depression	Eyes	TOM
Thompson et al., 2012/13	30	CAARMS	FEP	Hinting task, Visual jokes, DANVA-2, MSCEIT, ANSIE	AR, TOM, AS, SP

SIPS = Structured interview for Prodromal Syndromes, SOPS = Scale of Prodromal Symptoms, CAARMS = Comprehensive Assessment of At Risk Mental States, Chron = chronic schizophrenia patients, FEP = first episode patients, FEIT = Facial Emotion Identification Test, FEDT = Facial Emotion Discrimination Test, EMODIFF = Penn Emotion Discrimination Task ATT = Abbreviated Trustworthiness Task, IPSAQ = Internal, Personal and Situational Attributions Questionnaire, MSCEIT = Mayer-Salovey-Caruso Emotional Intelligence Test, TASIT = The Awareness of Social Inference Test, RAD = Relationships Across Domains Test, DANVA-2 = Diagnostic Assessment of Non Verbal Accuracy, ANSIE = Adult Nowicki Strickland Internal External Scale, AR = affect recognition, AS = attributional style, TOM = theory of mind, SP = social perception.

### Social cognition and transition to psychosis

Nine studies reported longitudinal data on the transition of UHR individuals to psychosis and the possible predictive value of social cognition for transition. Four studies examined Theory of Mind, four articles examined affect recognition, one study investigated attributional style and one study examined social perception (trustworthiness task). Not enough data were available to perform a meta-analysis to compare social cognitive performance between converters and non-converters across studies. We evaluated the studies qualitatively.

Three out of the four studies on Theory of Mind [45, 65, 66] did not find significant differences on baseline data between converters and non-converters after controlling for symptoms, IQ and age. This was the case for verbal (Strange Stories task, VSIT) and visual (Cartoon task, Eyes task) TOM tasks. This indicates that TOM does not predict transition into psychosis and is not useful as marker for the prediction of onset. One study on TOM [54] did find that a verbal TOM task (False Belief task) predicted conversion, however converters in this study had a significant lower IQ score than nonconverters and the control group was better educated than the UHR group.

Two [44, 49] out of three studies that investigated the difference in prosodic affect recognition between converters and non-converters at baseline did not find a significant effect. The third study [37] did not include enough participants that fulfilled the prosodic affect recognition task (2 converters) to be able to analyze the effect. Two studies [44, 67] did not find a significant difference on facial affect recognition and discrimination at baseline between converters and non-converters. However, one study [37] did find a clear difference for affect recognition as well as affect discrimination. Whereas there was no difference between the total at risk group and the control group, the authors did find a significant difference between converters and non-converters/controls. Converters where less accurate in the recognition of fear and anger. They labeled more emotional faces as ‘neutral’ in comparison to the non-converter group. A second study [49] found a significant difference between converters and non-converters in identifying neutral and fearful faces. In this study, converters mislabeled neutral faces as fearful, after controlling for symptoms, functioning and age.

A study on social perception [45] and attributional style [68] did not find any difference between converters and non-converters at baseline.

### Overall difference social cognition in UHR versus healthy controls

**Fig 2:**
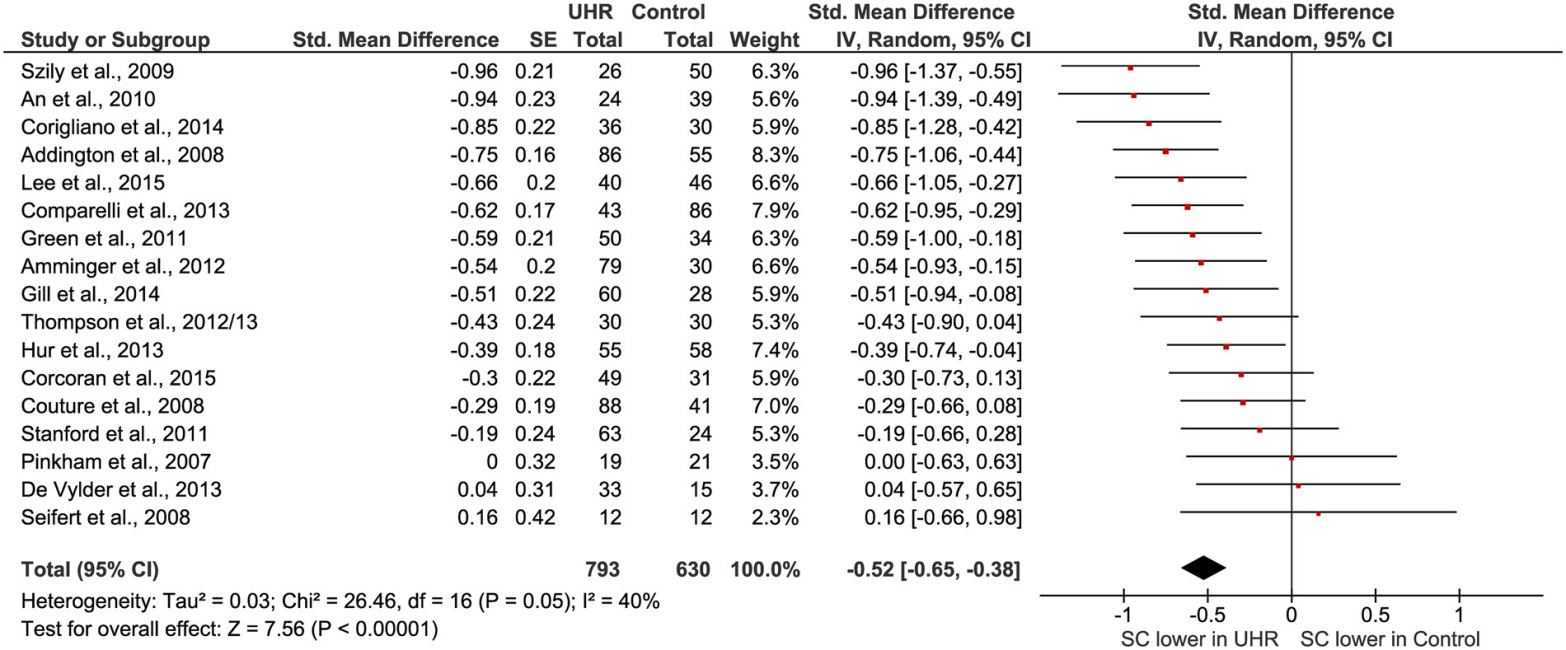
Forest plot of social cognition in UHR versus healthy controls.

The significant mean weighted effect size of all seventeen studies on the difference in social cognition in UHR versus healthy controls is 0.52 (95% Cl = 0.38–0.65). This is a medium effect size, according to Cohen’s nomenclature and this effect size is significant (Z = 7.56, p < 0.00001). The forest plot of this analysis is represented in Fig 2. Publication bias is not likely, as revealed by the Eggers test (F = 2.26; P>0.1). The Chi-square test was statistically significant and indicates heterogeneity; results must be interpreted with caution. The heterogeneity may be due to moderating factors, we intended to identify these possible factors using a moderator analysis (next paragraph). Another possible explanation for the heterogeneity may be the inclusion of several different constructs of social cognition in this meta-analysis. We therefore proceeded by splitting the studies into four subgroups based on previous literature: Affect recognition/discrimination, Theory of Mind, Attribution and Social perception/knowledge.

### Effect of moderators

The results of the moderator analyses are shown in Table 2. All effect sizes remained significant. The median split for age was 20 years and for number of participants the split was set on 50 participants. No significant moderator effects were found for age, number of subjects and gender. Unfortunately the moderating effect of IQ could not be studied due to the small number of studies reporting sufficient data.

**Table 2:** Moderator analyses.

Variabele	k	N (UHR)	Cohen’s D	95% CI	Chi^2^ within	Chi^2^ between
Age (mean years)						
<20	9	529	0.49 sign	0.34–0.64	9.31 ns	0.21 ns
>20	8	264	0.55 sign	0.31–0.80	16.40 sign	
Gender						
> 50% women	6	233	0.61 sign	0.39–0.84	8.25 ns	1.11 ns
> 50% men	11	560	0.46 sign	0.30–0.63	26.46 ns	
Number subjects						
<50	10	312	0.53 sign	0.31–0.75	19.85 sign	0.08 ns
>50	7	481	0.49 sign	0.35–0.64	5.92 ns	

### Affect recognition and discrimination

**Fig 3:**
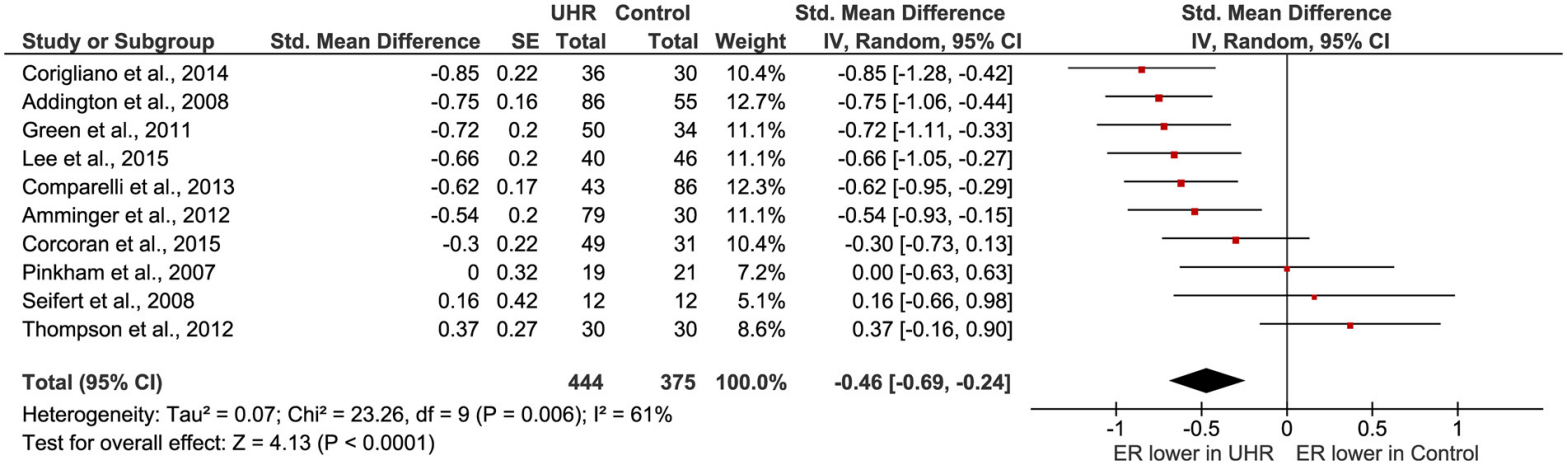
Forest plot of affect recognition in UHR versus healthy controls.

Ten studies reported on affect recognition in UHR participants and healthy controls (Table 1). All ten studies investigated the recognition of emotion in faces; three articles also examined the ability to recognize emotions in voices [37, 55, 69]. Affect recognition was the primary outcome in all articles except for Seifert et al. [70] who used the task in an fMRI study. All studies investigated affect recognition (to be able to name the appropriate emotion when a facial expression is shown), three studies also investigated affect discrimination (the ability to see if two facial expressions show the same or a different emotion) [37, 67, 71]. For the calculation of the total effect size, subtests where combined into one mean effect size before they were entered into the analysis to ensure independent observations [37, 55, 67, 69–72]. The significant (Z = 4.13; P < 0.0001) mean effect size of the studies was medium according to Cohen’s nomenclature (d = 0.46, 95% Cl = 0.24, 0.69) (Fig 3), with lower affect recognition in the at risk group. The Chi-square test was significant, which indicates that substantial heterogeneity is present. When we omitted the discrimination tasks and only included the ten recognition tasks, the significant mean effect size (Z = 4.21; P<0.0001) was approximately the same (d = 0.47, 95% Cl = 0.25, 0.69), with lower affect recognition in the at risk group. The Chi-square test was still significant. Only three studies investigated affect discrimination, therefore we did not calculate the effect size of this subgroup. Two out of three of the studies investigated if patients could see if two faces showed the same or a different emotion, and both studies did not find a significant difference between UHR patients and healthy controls [67, 71]. The third study [37] took a slightly different approach and investigated if participants could discriminate which of two faces showed a more intense expression of the same emotion. This study did find a significant difference between the healthy controls and the UHR patients. More research into emotion discrimination is needed.

When we looked at the facial affect recognition tasks only, the significant effect size (Z = 4.47; P<0.00001) stayed approximately the same (d = 0.48, 95% Cl = 0.27, 0.69), with lower facial affect recognition in the UHR group. The Chi-square test was still significant. Only three studies [37, 55, 69] reported on prosodic emotion recognition, which is not enough to calculate a mean effect size. All three articles found a significant lower score on prosodic emotion recognition in UHR participants in comparison to healthy controls. More research is needed on this subject.

### Theory of mind

**Fig 4:**
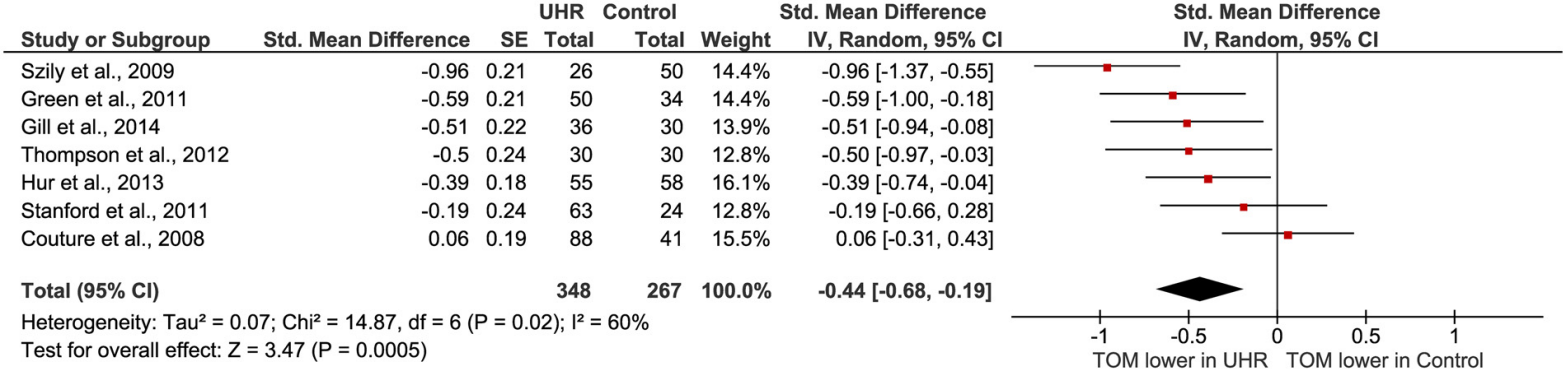
Forest plot of Theory of Mind in UHR versus healthy controls.

Seven studies investigated Theory of Mind (TOM) in participants in the UHR phase (Table 1). TOM was the primary outcome for all studies. Verbal tasks (False Belief task, Strange Story task, the Awareness of Social Inference task and Hinting task) as well as visual tasks (Cartoon task, Reading the Eyes task, Visual Jokes task) were used in the studies. Multiple tests within one study where combined into one mean effect size before entering into the analysis to ensure independent observations [56, 66, 69]. The significant (Z = 3.47; P = 0.0005) mean effect size of the studies was medium according to Cohen’s nomenclature (d = 0.44, 0.19, 0.68) (Fig 4). The Chi-square test for homogeneity was significant, which indicates the presence of considerable variability in outcome. This may be caused by clinical and methodological diversity of the studies. The results must therefore be interpreted with caution. We performed a meta-analysis on the subgroups verbal TOM and visual TOM. The medium (d = 0.52; 95% Cl = 0.30, 0.74) total effect size of the five verbal TOM tasks was significant (Z = 4.61, P = 0.00001) and the Chi-square test was not significant. The mean effect size of the five visual TOM tasks was medium (d = 0.33, 95% Cl = 0.03, 0.68) and not significant (Z = 1.83; P = 0.07). The Chi square test was significant for this subsample.

### Attribution style

Only three studies where found on attribution style and individuals in the UHR phase (Table 1). Because of the low amount of studies on this subject, we did not calculate a pooled effect size but investigated the studies separately. Thompson et al. [73] and DeVylder et al. [68] investigated the extent in which individuals tended to attribute the cause of an event to external causes (externalizing bias). An et al. [74] and DeVylder et al. [68] investigated the extent in which individuals tended to attribute the cause of a negative event to other people (personalizing bias). For both the externalizing and the personalizing bias, one study found a significant effect and the other study did not find any effect. Clearly, more research is needed on this subject before any conclusions can be made.

### Social perception / social knowledge

Three studies investigated social perception/knowledge in UHR participants (Table 1). All studies used very different measurement instruments and examined different elements of social perception/knowledge: managing emotions and the emotions of others in social situations [69], how to estimate the trustworthiness of strangers [75] and how to understand social relationships, based on relational models theory [76]. Because of the low amount of studies on this subject and the diversity of the used measurement scales, we did not calculate a pooled effect size but investigated the studies separately.

## Discussion

In addition to previous work [20, 35] we performed a meta-analysis of published studies to examine the evidence regarding impaired social cognition in individuals at risk of psychosis. We chose to only include studies that defined at risk patients using UHR criteria of Yung and McGorry [3], too ensure a homogeneous sample. In agreement with previous studies [20, 35] we found evidence for significant impairment of social cognition in the UHR group, with effect sizes in the moderate range, which is comparable to the severity of other cognitive deficits in participants in the UHR phase [4, 17, 77]. There was substantial heterogeneity of effect sizes. No moderator effect was found for age, gender or sample size. Not enough data were available to calculate the possible moderator effect of IQ.

Sub analyses for affect recognition showed that UHR participants demonstrate significant moderate deficits in affect recognition in faces. Not enough studies were published to perform an analysis of prosodic affect recognition tasks, but the three studies that were found all reported problems in recognizing emotions in voices for UHR participants in comparison to controls. More research on this subject is needed. Furthermore, sub analyses showed that UHR patients have problems with *recognizing* emotions, whereas it is not clear if they have the same difficulties in *discriminating* between twee different emotions. The two studies that were found on this subject did not find a significant difference between the UHR group and controls. A possible explanation for this result can be that the emotion discrimination task is less difficult than the emotion identification task [78]. It is possible that people at risk of psychosis do not experience difficulties in discriminating between two different emotions, however they possibly do have difficulties in differentiating between intense or less intense versions of the same emotions, as one study found (Corcoran et al. 2015). More research is needed on this subject.

Sub analyses of TOM showed significant problems with verbal TOM tasks in UHR participants in comparison to healthy controls. A moderate effect was found for visual TOM, but this effect was not significant. This is not in line with a previous meta-analysis on TOM in participants in the UHR phase [34], where significant moderate effect sizes for visual TOM where found (d = 0.40, 95% Cl = 0.14–0.70; Z = 2.94, P = 0.003). The same articles as used in this meta-analysis were used in their study, except for Kim et al. [54]. This article is the precursor of Hur et al. [56] that we included. The samples were drawn from the same participant pool in both articles, but Hur included more participants resulting in a higher weight of the results of this study. Bora et al. also used data from Ohmuro et al. [79] that were presented at a conference, while conference data did not meet our inclusion criteria. A lack of power might be accountable for the absence of a significant effect for visual TOM in our study, given that the pooled ES approached significance (P = 0.07). Another possible explanation for the difference in outcome in verbal and visual TOM is that verbal TOM tasks require more cognitive effort in comparison to visual TOM tasks. Verbal tasks ask for the ability to process a lot of verbal information such as large amounts of written texts. These tasks also correlate highly with general cognitive functioning [56]. Thus verbal TOM tasks might be challenging for people in the UHR phase due to problems in general cognitive functioning. More research is needed on visual TOM tasks to determine if only verbal TOM is affected.

Three studies where found regarding attributional style in participants in the UHR phase, with different results. For both the externalizing and the personalizing bias, one study found a significant effect and the other study did not find any effect. A possible explanation for these different outcomes is that the studies used different measurement instruments and measured different constructs of attributional style. Clearly, more research is needed on this subject before any conclusions can be made.

All three included studies on social perception used very different measurement instruments and examined different elements of social perception/knowledge. Therefore we decided not to calculate a pooled effect size for this subdomain. As both social perception and social knowledge are severely impaired in patients with schizophrenia [80], further research is needed on this subject to investigate the extent and nature of impairments in this domain for individuals in the UHR phase.

A recent meta-analysis in social cognition in people with schizophrenia [80] revealed large effects on emotion perception and TOM, among others. This may suggest that UHR groups perform better than schizophrenia patients, but worse than healthy controls as would be expected on the basis of previous findings regarding cognitive functioning and UHR participants [81].

Not enough data were available to perform a meta-analysis to compare social cognitive performance between converters and non-converters across studies. We evaluated the studies qualitatively. Verbal as well as visual Theory of Mind did not seem to predict transition into psychosis, as three out of four studies did not find any differences between converters and non-converters at baseline after controlling for symptoms, IQ and age. The one study that did find a verbal TOM task to predict conversion [54] did not control for IQ and converters had a significant lower IQ score than non-converters at baseline. The lower performance of the converters in this study may therefore be caused by lower general cognitive function instead of specific TOM problems, as the verbal information processing that is required for verbal TOM tasks may be challenging for participants with lower IQ scores.

First studies on prosodic affect recognition as a predictor of transition found no significant differences between converters and non-converters at baseline. Results on facial affect recognition are less clear. Most studies did not find any difference between converters and non-converters on overall affect recognition in faces. However, two studies showed significant problems of converters with recognizing specific emotions, in particular fear and anger, in comparison to non-converters at baseline. More studies are needed on this subject and the possible problems with the recognition of specific emotions need to be taken into account.

The two studies on attributional style and social perception did not find any significant differences between converters and non-converters at baseline. More longitudinal studies are needed to clarify the possible predictive value of these domains of social cognition for transition.

With the current quantitative integration of published findings, we aimed to investigate whether the four social cognitive subdomains as identified by Pinkham et al. [23] are impaired in the UHR phase. Pinkham et at al. additionally highlighted ‘empathy’ as an area of importance within the field of social cognition and psychosis, but did not nominate this construct as a separate domain of social cognition, due to the possible overlap of the cognitive elements of empathy with Theory of Mind. As many authors present empathy as a separated construct [82] and as several instruments measure empathy specifically [83, 84], additional research is needed at a fundamental level to identify the possible overlap and unique elements of these domains of social cognition.

A number of limitations of our study should be noted. Since only three studies on attributional style and four very different studies on social perception were found, more research is needed on these subjects before any conclusions can be drawn on impairment in the UHR phase. More research on the association of impaired social cognition in the UHR phase with actual transition into psychosis is needed to determine the clinical consequences of impaired social cognition in the UHR phase.

There is some evidence for social cognition to be significantly influenced by IQ [56, 66]. It would therefore be useful to compare studies that include IQ or education level into the analysis as a covariate with studies that do not include these factors as covariates. Unfortunately, the necessary data to perform this analysis (F value or d after correction for IQ) was not provided in the articles included in this study. The studies that did include IQ as a covariate did not find a different effect before and after correction for IQ. Further research is needed on this subject.

A general limitation of meta-analysis is the dependence on methodology of published studies. The diversity in measurements used in the studies was large and the psychometric properties of most of the used instruments are unknown. As Pinkham et al. [23] have stated; there is a clear need for studies designed to assess the psychometric properties of social cognition measurement instruments.

## Conclusion

This meta-analysis reveals consistent impairments in social cognition in people in the UHR phase, in comparison to healthy controls. Significant deficits are detected in affect recognition and verbal TOM. We did not calculate the effect sizes for social cognition/knowledge and attributional style as more studies are needed for these subdomains. No moderator effects were found for age, gender and sample size. A majority of studies did not find a correlation between social cognition deficits and transition to psychosis, after controlling for IQ, education and baseline symptoms, which may suggest that social cognition in general is not a useful marker for the development of psychosis. However some studies suggest the possible predictive value of more specific forms of social cognition, in particular verbal TOM and the recognition of specific emotions in faces for the transition into psychosis.

## Supporting Information

S1 Checklist PRISMA checklist for meta-analyses. (DOC)
